# Estimation of High-Speed Liquid-Jet Velocity Using a Pyro Jet Injector

**DOI:** 10.1038/s41598-019-56511-x

**Published:** 2019-12-27

**Authors:** Naohisa Takagaki, Toru Kitaguchi, Masashi Iwayama, Atsushi Shinoda, Hiroshige Kumamaru, Itsuro Honda

**Affiliations:** 1 Department of Mechanical Engineering, University of Hyogo, Himeji, 671-2280 Japan; 2 Medical Device Division, Medical Device Research Center, Daicel Corporation, Toyonaka, Osaka 560-8531 Japan; 3 Corporate Research Center, R&D Headquarters, Daicel Corporation, Himeji, Hyogo 671-1283 Japan

**Keywords:** Mechanical engineering, Fluid dynamics

## Abstract

The high-speed liquid-jet velocity achieved using an injector strongly depends on the piston motion, physical property of the liquid, and container shape of the injector. Herein, we investigate the liquid ejection mechanism and a technique for estimating the ejection velocity of a high-speed liquid jet using a pyro jet injector (PJI). We apply a two-dimensional numerical simulation with an axisymmetric approximation using the commercial software ANSYS/FLUENT. To gather the input data applied during the numerical simulation, the piston motion is captured with a high-speed CMOS camera, and the velocity of the piston is measured using motion tracking software. To reproduce the piston motion during the numerical simulation, the boundary-fitted coordinates and a moving boundary method are employed. In addition, we propose a fluid dynamic model (FDM) for estimating the high-speed liquid-jet ejection velocity based on the piston velocity. Using the FDM, we consider the liquid density variation but neglect the effects of the liquid viscosity on the liquid ejection. Our results indicate that the liquid-jet ejection velocity estimated by the FDM corresponds to that predicted by ANSYS/FLUENT for several different ignition-powder weights. This clearly shows that a high-speed liquid-jet ejection velocity can be estimated using the presented FDM when considering the variation in liquid density but neglecting the liquid viscosity. In addition, some characteristics of the presented PJI are observed, namely, (1) a very rapid piston displacement within 0.1 ms after a powder explosion, (2) piston vibration only when a large amount of powder is used, and (3) a pulse jet flow with a temporal pulse width of 0.1 ms.

## Introduction

High-speed water-jet techniques are extremely useful in broad industrial fields, for example, the cutting of metallic boards, wooden boards, concrete blocks, vegetables, and human organs^1–3^. Such a high-speed water jet is generally produced by high-pressure gas, a piezo actuator, a metallic spring, or a falling or accelerated weight. Many driving forces used to generate a water jet have been applied in fundamental studies, and an adequate driving force has been chosen in each industrial field.

The needs regarding the water-jet properties (e.g., the jet velocity, duration time, and diameter, and the liquid type) vary for each field. Several previous studies have focused on the water jet properties both experimentally and numerically^4–9^. Previously, some studies (e.g. Schramm and Mitragotri^4^) measured the mean water-jet velocity and container pressure by a load cell. Recently, a high-speed photography^5–7^ and laser Doppler velocimetry^8^ have been demonstrated as options for jet-velocity measurement. However, there are few reports of numerical simulation^9^ for predicting the high-speed jet velocity because it is very difficult to execute a numerical simulation with the subroutines for the mobile interface, multiphase flow, and compressible flow simultaneously. For driving forces, new types of jet injectors have recently been developed. Tagawa *et al*.^5^ and Moradiafrapoli and Marston^6^ demonstrated a microjet provided by a laser-induced cavitation in a container. In addition, Taberner *et al*.^7^ developed a Lorentz-force jet injector. Because these studies^5–7^ aim to develop new needle-free jet injectors, they investigated the relationship between the jet velocity and the penetration depth of the liquid jet into gelatin or soft material. However, because the jet velocity is quite high and the application fields vary, there are few studies investigating the universal mechanism of liquid jet ejection.

The purpose of the study is therefore to investigate the liquid-jet ejection mechanism through laboratory experiments and numerical simulations and to propose a universal fluid dynamic model for estimating the liquid-jet ejection velocity.

## Liquid Compressibility

The liquid pressure increases rapidly due to high pressurization when using a liquid-jet injector. Thus, we first revisit an equation-of-state for a liquid and its compressibility. As an equation-of-state for a compressible liquid, the Tait equation-of-state was previously proposed, which is written as follows:

1 $$Q={Q}_{0}-\frac{C{\log }_{10}(B+P)}{B+{P}_{o}},$$

where *Q* is the liquid volume under high pressure, *Q*_0_ is the liquid volume under ordinary temperature and pressure, *P* is the liquid pressure, *P*_0_ is the ordinary pressure (*P*_0_ = 0.1 MPa), *B* is an empirical constant (in bar)^10^, and *C* is an empirical constant (in ml/g)^11^. The values of *B* and *C* are represented in the following equations:

2 $$B=2.668+1.9867T-0.311{T}^{2}+\frac{1.778}{1000}{T}^{3},$$

3 $$C=0.3150{Q}_{0},$$

where *T* is the liquid temperature. The following Murnaghan–Tait equation-of-state, which is a revised version of the Tait equation-of-state, is commonly used:

4 $${(\frac{\rho }{{\rho }_{0}})}^{n}={(\frac{{Q}_{0}}{Q})}^{n}=\frac{K}{{K}_{0}},$$

where *ρ* is the liquid density under high pressure, *ρ*_0_ is the liquid density under ordinary temperature and pressure, *n* is the density constant, *K* is the bulk modulus under high pressure, and *K*_0_ is the bulk modulus under ordinary pressure. In a study conducted by Richardson *et al*.^12^, the values of *n* vary from 6.9 to 7.4 with a temperature range of 20–60 °C and a pressure range of 5–250 MPa (see Table IX^11^). Richardson *et al*.^12^ calculated the average rounded value of *n* = 7.15, which is also the default value used in ANSYS/FLUENT. Thus, we used *n* = 7.15 in the present study. Although *K*_0_ varies from 1.9 to 3.1 GPa within a temperature range of 0–50 °C and a pressure range of 0.1–150 MPa according to a mechanical engineering handbook^13^, a constant value of *K*_0_ = 2.2 GPa was used in the present study, which is also the default value in ANSYS/FLUENT. The relationship between *K* and *K*_0_ is represented specifically through the following equation:

5 $$K={K}_{0}+n\Delta P,$$

where Δ*P* is the pressure change (Δ*P* = *P* − *P*_0_). In the present study, we used the Murnaghan–Tait equation-of-state for calculating the liquid volume and density under high pressure.

The fluid compressibility is represented using the Mach number, *Ma*, as follows:

6 $$Ma=\frac{U}{c},$$

where *U* is the fluid velocity and *c* is the speed of sound in a fluid. The value of *c* is determined as follows:

7 $$c=\sqrt{\frac{K}{\rho }}.$$

Using Eqs. (4) and (5) and *ρ*_0_ = 998.2 kg/m^3^, Eq. (7) is expanded as

8 $$c={\rho }_{0}^{-\frac{1}{2}}{K}_{0}^{\frac{1}{2n}}{K}^{\frac{1}{2}-\frac{1}{2n}}=0.142{(2.2\times {10}^{9}+7.15(P-{10}^{5}))}^{0.43}.$$

Therefore, using Eqs. (6) and (8), and assuming that *U* is lower than 450 m/s, all values of *Ma* under higher than ordinary pressure are lower than 0.3, which is the criterion for separating the fluid compressibility; that is, *Ma* < 0.3 indicates an incompressible fluid (low Mach approximation), and *Ma* > 0.3 indicates a compressible fluid. This simple estimation clearly shows that an incompressible liquid condition (low Mach approximation) can be acceptable under high pressure in water if *U* is lower than 450 m/s. Note that the present experimental and numerical conditions satisfy this incompressible condition, except for Run 6 (see the Results and Discussion section). In addition, the numerical simulations consider the change in liquid density variation but do not solve the energy equation (see the Numerical Procedure of ANSYS/FLUENT section).

## Experiment Methods

**Figure 1:**
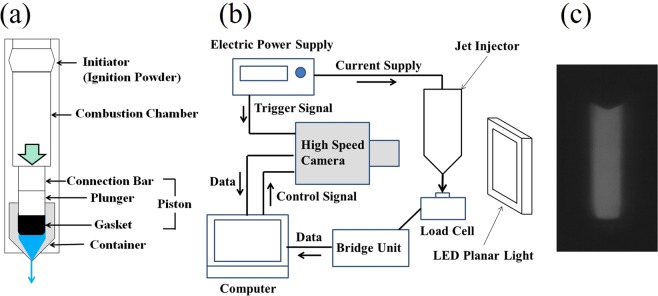
Schematic of the experimental apparatus: (**a**) pyro jet injector (PJI), (**b**) measurement equipment, and (**c**) image of the container. In (**c**), the upper triangle part shows the gasket’s edge, and the white area shows the container, as recorded by the high-speed camera at Run 6 and *t* = 0.79 ms.

Figure 1 shows the experiment apparatus and the pyro jet injector (PJI), developed at Daicel Co., Japan^14–18^. The device was constructed using a combustion chamber, piston, and container, and the shape was cylindrical (see Fig. 1a). The combustion chamber used an ignition powder (IP), by which no gas was generated during the combustion. The combustion products generated in the combustion chamber were completely sealed with O-rings, and no combustion products leaking out of the chamber were detected. An explosive containing zirconium and potassium perchlorate was used as the IP. The zirconium and potassium perchlorate burned at high temperature and pressure immediately after ignition, although the generated pressure subsequently decreased suddenly because no gas component was available when ordinary temperatures were supplied and the combustion products were condensed. The powders were manufactured in-house.

The piston was constructed using a steel connection bar, plunger, and gasket. The PJI was 0.222 m in length and 0.11 m in width. The container was made from transparent polycarbonate (PC); the total inner length of the container was 0.172 m and the diameter (*D*_C_) was 4.65 mm. The container was filled with distilled water, and the water volume was 100 mm^3^, which corresponds to an initial longitudinal liquid length *L*_C0_ of 5.5 mm. The distilled water was sealed with a piston with a rubber gasket. The bottom of the container was tapered with a straight cylindrical nozzle with a diameter *D*_N_ of 0.1 mm and a length *L*_N_ of 1.0 mm. The laboratory experiments were conducted in six runs, each using a different IP weight. The device operated as follows: The IP was ignited by an overloaded current in the combustion chamber, which was controlled using an electric power supply (ADCMT 6242). The connection bar was accelerated by the pressure generated from the IP combustion. The connection bar then slid the plunger into the container. The liquid extruding into the container by the piston was released at a high pressure from the nozzle.

When the explosion occurred, the filled water was ejected from the bottom nozzle at an extremely high velocity. To capture images of the piston motion after the explosion, we used an LED planar light (CCS TH2-100 × 100) as a background light, a high-speed CMOS camera (Photron FASTCAM SA-X2), close-up rings (Nikon PK-11A, PK-12, PK-13), and a lens (Nikon, AI Micro-Nikkor 105 mm f/2.8 S). We visualized the motion of the piston using the background light (see Fig. 1b). The timing for the captured images was controlled with an external trigger signal from the electric power supply (ADCMT 6242), which was used for IP ignition. Images (see Fig. 1c) of 5.43 mm × 13.89 mm (200 pixels × 512 pixels) were captured at a frame rate of 100,000 fps and with a shutter speed of 0.8 μs. The spatial resolution was 9.3 μm/pix. More than 1,000 images were captured. Software (Photron PFA) was used to estimate the piston displacement with a motion tracking method. The measurement was performed at a controlled room temperature ranging from 24 to 26 °C.

The ejection velocity of the liquid jet was indirectly measured using a load cell (Kyowa Electronic Instruments, LMA-5N). A schematic of the experimental setup is shown in Fig. 1b. The load cell was set vertically 0.5 mm below the nozzle of the PJI. The load cell was connected to a bridge unit (Kyowa Electronic Instruments, DBU-120A). When the liquid jet impacted the load cell, the force (*F*_LC_) acting on the load cell was measured and recorded. The liquid-jet ejection velocity *U*_N_ was estimated using the momentum balance on the load cell, namely,

9 $${F}_{LC}=\rho {A}_{N}{U}_{N}^{2},$$

assuming that the jet has the same velocity (*U*_N_) and cross-sectional area (*A*_N_) as at the nozzle of the PJI. Thus, because ${A}_{N}=\pi {D}_{N}^{2}/4$, *U*_N_ can be calculated as follows:

10 $${U}_{N}=\sqrt{\frac{4{F}_{LC}}{\rho \pi {D}_{N}^{2}}}.$$

## Numerical Procedure of ANSYS/FLUENT

A two-dimensional numerical simulation with an axisymmetric approximation was conducted using the commercial software, ANSYS/FLUENT (ver. 18.1)^19,20^ for reproducing the piston motion and liquid ejection. The governing equations for an incompressible Newtonian liquid flow are given by an equation of continuity and the Navier–Stokes (N–S) equation, which are expressed as follows:

11 $$\frac{\partial }{\partial t}\rho +\nabla \cdot (\rho {\boldsymbol{u}})=0,$$

12 $$\frac{\partial }{\partial t}\rho {\boldsymbol{u}}+\nabla \cdot (\rho {\boldsymbol{uu}})=-\,\nabla P+\nabla \cdot (\mu \nabla {\boldsymbol{u}})+\rho {\boldsymbol{g}},$$

where *ρ* is the liquid density, ***u*** is the liquid velocity vector, *P* is the pressure, *μ* is the liquid viscosity, and ***g*** is the acceleration vector due to gravity (*g* = 9.80665 m/s^2^). The direction of acceleration from gravity is positive in the *x*-direction. To consider the variation in liquid density, we employed the Murnaghan–Tait equation-of-state as follows:

13 $${(\frac{\rho }{{\rho }_{0}})}^{n}={(\frac{{Q}_{0}}{Q})}^{n}=\frac{K}{{K}_{0}},$$

the details of which are given in the Liquid Compressibility section. Because the compressibility in the present numerical conditions is low, a low Mach-number approximation is employed, and it is not considered necessary to solve the energy equation simultaneously.

**Figure 2:**
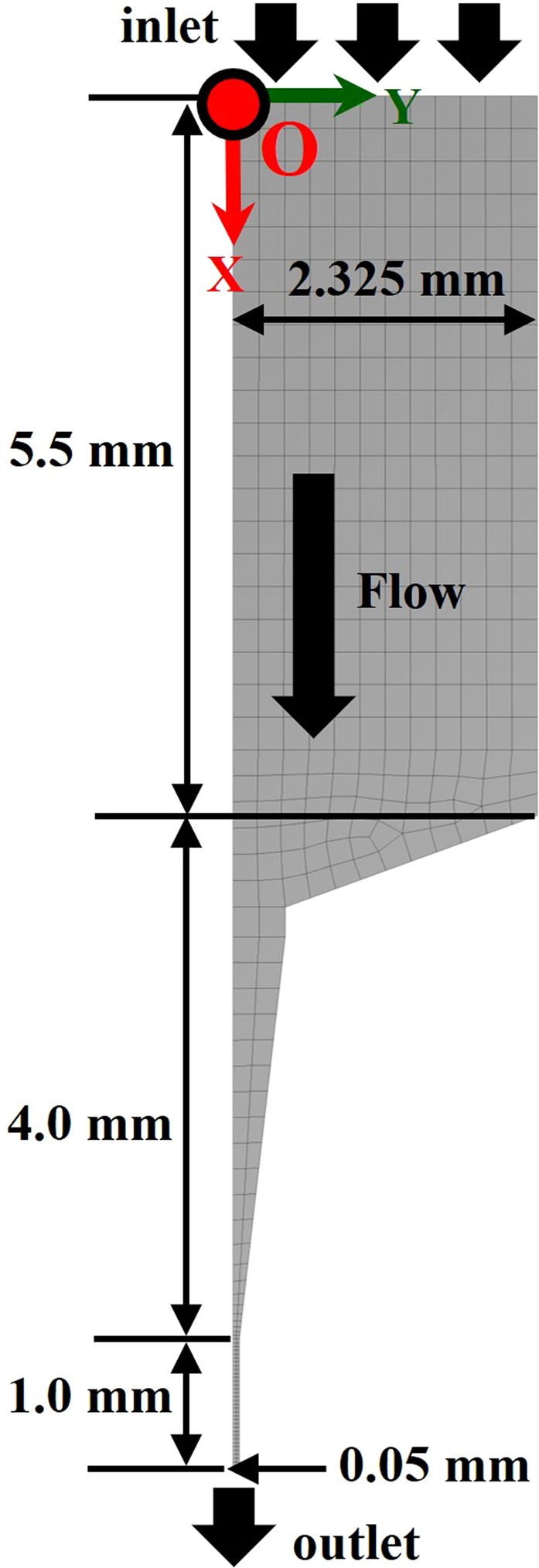
Two-dimensional computational domain and grids. Grids are depicted coarsely, and actual grid spacings in both the *x-* and the *y-*directions are 40 μm in the container and 5 μm in the nozzle during all runs. The red circle with the character “O” indicates the origin.

The computational domain and numerical grids for the computations of a liquid flow are shown in Fig. 2. Note that the gray regions in the figure indicate the liquid region. In the figure, the fluid flows from top to bottom. The size of the computational domain is (*L*_C0_ + *L*_T_ + *L*_N_) × *R*_C_ in the streamwise (*x*) and spanwise (*y*) directions, where *L*_C0_ is the initial longitudinal length of the liquid (*L*_C0_ = 5.5 mm), *L*_T_ is the taper length (*L*_T_ = 4.0 mm), *L*_N_ is the nozzle length (*L*_N_ = 1.0 mm), and *R*_C_ is the container radius (*R*_C_ = *D*_C_/2 = 2.325 mm). The origin (*x* = *y* = 0) is located at the initial position of the piston and centerline (see Fig. 2). The grid was constructed by a boundary-fitted curvilinear coordinate and 11,822 quad elements during all runs. The grid spacings in both the *x-* and the *y*-directions were 40 μm in the container and 5 μm in the nozzle during all runs.

To reproduce the mobile wall of the piston, we employed the moving boundary method (“dynamic meshing” option) in ANSYS/FLUENT. As the input data of the piston wall displacement during the numerical simulation, the displacement data measured from the laboratory experiments (see the Experiment Methods section) were used. An outflow boundary condition with a constant gauge pressure was applied at the outlet of the nozzle. A nonslip boundary condition was applied at the top and side boundaries, including at the walled parts of the piston. A slip boundary condition was applied at the centerline.

For the initial conditions of the flow field, still liquid with a density of 998.2 kg/m^3^ was set, which corresponds to the water density at approximately 20 °C. In addition, the kinetic viscosity of water *ν* was set to be a constant value of 1.0 × 10^−6^ m^2^/s throughout the simulations. This value corresponds to the water kinetic viscosity at approximately 20 °C. The constant values of the density constant (*n* = 7.15) and the bulk modulus under ordinary pressure (*K*_0_ = 2.2 GPa) were used (see the Liquid Compressibility section).

The governing Eqs. (11–13) were discretized to construct the finite volume formulation, and the pressure-implicit with splitting of operators (PISO) algorithm was used to solve the N–S equation. For spatial discretization in the N–S equation, the gradient was analyzed using least squares cell-based gradient evaluation, the pressure was discretized through a standard scheme, and the momentum was discretized using a second-order upwind scheme. The time integration was conducted using an implicit first-order method. The viscous term in the N–S equation was solved using the eddy viscous model assigned by the realizable k–ε model in ANSYS/FLUENT for all runs. The time increment *dt* was fixed at 1 μs, and the total step time was 2,000 during all runs. The central processing unit (CPU) time was approximately 3–5 h using a single core for over 2,000 steps (2 ms) on a computer at Daicel Co.

## Results and Discussion

### Piston displacement

**Figure 3:**
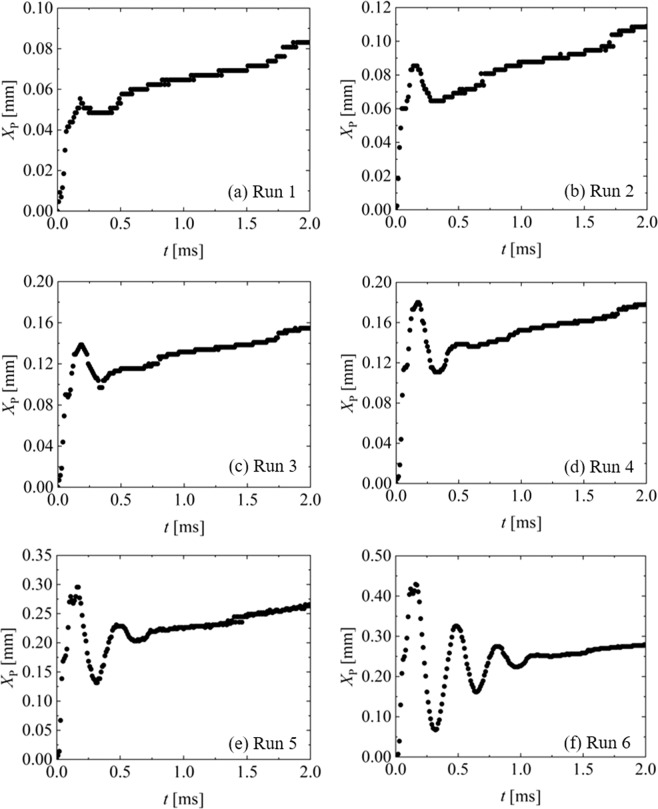
Time changes of piston displacement. The 95% confidential intervals for measured displacements were 0.0044, 0.021, 0.0085, 0.0078, 0.045, and 0.029 mm for Runs 1–6, respectively.

Figure 3 shows the time change of the piston displacement. Here, the difference between the experimental runs indicates the different IP weights. In addition, the measurements were performed thrice in each run. The 95% confidential intervals for the measured displacements were 0.0044, 0.021, 0.0085, 0.0078, 0.045, and 0.029 mm for Runs 1–6, respectively. Thus, the ensemble-averaged measurement error was approximately 12%, which indicates good repeatability for measuring the piston displacement.

During Run 1 (Fig. 3a), the piston rapidly moves forward at *t* = 0–0.2 ms. The maximum piston velocity is approximately 4 m/s. The peak occurs at *t* = 0.2 ms, and then regresses. After *t* = 0.5 ms, the piston advances again. The piston achieves a uniform linear motion after *t* = 1.0 ms. It takes approximately *t* = 400 ms for the piston to stop at the end of the container, which is not indicated in the enlarged display of the initial displacement ranging from 0 to 2 ms in Fig. 3(a). The trends of the piston displacement during Runs 2 and 3 (Fig. 3b,c) are similar to those in Run 1. However, as shown in Fig. 3(d–f), several peaks occurred at approximately *t* = 0.2–1.2 ms during Runs 4–6. The vibration of the piston might be due to 1) the resonance in the combustion chamber or 2) the balance between the pressure in the combustion chamber and that in the container. The detailed mechanism of possibility 2 is described below: After ignition, the pressure (*P*_CC_) in the combustion chamber increases, and *P*_CC_ is higher than the pressure (*P*_C_) in the container. Thus, the piston advances due to the pressure balance. Subsequently, *P*_C_ increases due to compression from the piston movement, and *P*_C_ becomes higher than *P*_CC_. This condition pushes the piston back.

These trends of the piston displacement imply that (1) an initial and extremely rapid displacement at 4 m/s might occur due to the impulsive force of the powder explosion and (2) the piston vibrates when the explosion is sufficiently strong. Rapid displacement with vibration is a common feature of jet injectors^6,7,21–24^. In particular, recent studies^23,24^ with a spring-type jet injector indicated that their piston moves rapidly and attains a maximum velocity of 0.8 m/s with vibration.

### Liquid-Jet ejection velocity

There have been some recent studies measuring a high-speed liquid jet velocity. One^6^ of the studies estimated the time-averaged initial jet velocity from image sequences of the jet by a high-speed camera. Another recent study^22^ also measured an instant jet velocity by a single cooled CCD camera and strobe and estimated the jet velocity from the plunger velocity. These studies confirm the difficulty of measuring the time-dependent high-speed jet velocity. Thus, in the present study, we predicted two jet velocities from the numerical simulation and a model (see the Fluid Dynamic Model section) along with the measurement of time-averaged initial jet velocity by a load cell.

**Figure 4:**
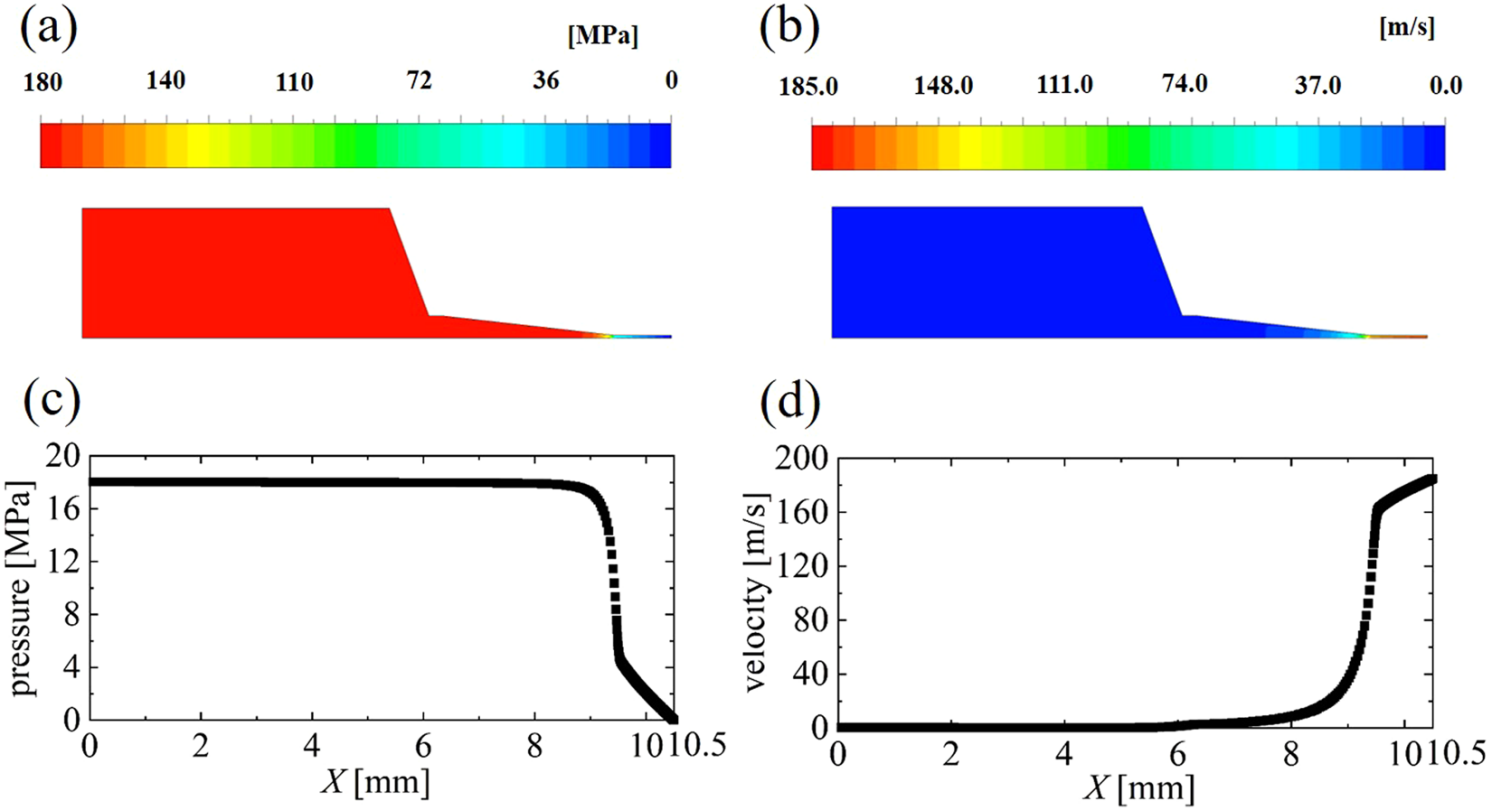
Instantaneous liquid pressure and velocity in the container during Run 1 and at the peak of the piston displacement (*t* = 0.2 ms, see Fig. 3a). (**a**) *x*-*y* distribution of the pressure, (**b**) *x*-*y* distribution of the velocity, (**c**) *x* distribution of the pressure at the centerline (*y* = 0 mm), and (**d**) *x* distribution of the velocity at the centerline (*y* = 0 mm).

Figure 4 shows the liquid pressure and velocity in the container during Run 1 and at the peak of the piston displacement (*t* = 0.2 ms, see Fig. 3a). From Fig. 4(a,b), we can see that the two-dimensional distributions of the pressure and velocity take high and low values, respectively, at the middle part of the container (*x* ~ 3.0 mm). The pressure is low at the nozzle part, and the velocity is high. This is due to the contraction of a thin nozzle. In addition, no separated flow occurs. Details of the streamwise distributions of the liquid pressure and velocity at the centerline (*y* = 0 mm) are provided in Fig. 4(c,d). From Fig. 4(c,d), the pressure and velocity take constant values from the surface of the piston wall (*x* = 0.0 mm) to the middle part of the nozzle (*x* = 9.0 mm). This indicates that there is no separation flow and no pressure drop both at the container and taper parts.

**Figure 5:**
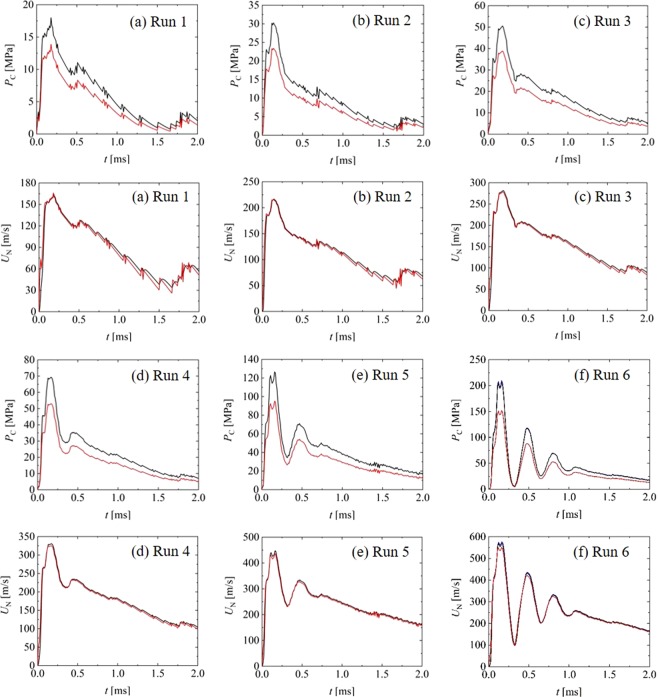
Time changes of pressure in the container and liquid-jet ejection velocity. Black and red bold curves show values predicted by ANSYS/FLUENT and the fluid dynamic model (FDM), respectively. In (f), black and blue bold curves show the values at 20 and 30 °C predicted by ANSYS/FLUENT, respectively.

Figure 5 shows the pressure *P*_C_ at the center point of the container and the liquid-jet ejection velocity *U*_N_. In the figure, the values predicted by ANSYS/FLUENT are shown as black curves, and the values estimated by the fluid dynamic model (FDM) are shown as red curves. Note that the FDM results are described in the Fluid Dynamic Model section. In ANSYS/FLUENT, *P*_C_ and *U*_N_ are calculated as the mean pressure at *x* = 2.75 mm and cross-sectional mean velocity at the outlet, respectively. As shown in Fig. 5(a), the pressure and ejection velocity rapidly increase due to the rapid displacement of the piston at *t* = 0–0.2 ms. A peak occurs at *t* = 0.2 ms, which corresponds to the peak piston displacement, and then regresses. Although the piston advances again after *t* = 0.5 ms, the pressure and ejection velocity take a secondary peak at *t* = 0.5 ms, after which they decrease. In addition, although the piston requires a motion similar to a linear uniform motion after *t* = 1.0 ms, the pressure and ejection velocity decrease. This is because the liquid compresses at *t* = 1.0 ms and relaxes after *t* = 1.0 ms. The trends of the pressure and ejection velocity during Run 1 are similar to those of Runs 2 and 3, as shown in Fig. 5(b,c). However, in Fig. 5(d–f), there are several peaks at approximately *t* = 0.2–1.2 ms during Runs 4–6. Such fluctuations in the pressure and ejection velocity are due to the piston vibration. In addition, such pulse jet flows with a temporal width of approximately 0.1 ms are specially generated through the pulse force owing to the pyro energy. To summarize, there is a possibility that these trends for the ejection velocity are due to (1) the impulsive force of the powder explosion, (2) the resonance in the combustion chamber, (3) the balance between the pressure in the combustion chamber and that in the container, and (4) a variation in the liquid density. In particular, the pulse jet flow has a characteristic temporal width of 0.1 ms, which is organized by the presented PJI, because other driving-type jet injectors do not have such a characteristic^4,25^.

In Run 6, the maximum ejection velocity of 550 m/s (and maximum pressure of 200 MPa) at *t* = 0.2 ms in Fig. 5(f) may be excessively high, and this condition does not satisfy the incompressible flow, where the threshold velocity is 450 m/s (see the Liquid Compressibility section). In the numerical simulation by ANSYS/FLUENT, the deformation (compliance) of the PC container is ignored. In addition, the liquid jet is not simulated after the liquid ejection from the needle’s outlet as a multiphase jet in ANSYS/FLUENT, which may affect the ejection velocity. Such dual effects of the container’s compliance and multiphase jet are very difficult to include in the present precise simulation. If we can consider such dual effects in the simulation, the maximum ejection velocity of 550 m/s should be suppressed because of the lower container’s pressure and higher pressure loss at the needle’s outlet than the present numerical simulation. More complicated precise numerical simulations, including the subroutines of the fluid-structure interaction (the container’s compliance) and multiphase flow, are needed in the future.

In the numerical simulation by ANSYS/FLUENT, the temperature change due to the water compression is also neglected; that is, the numerical simulation executed under the approximation of the isothermal change along with the Murnaghan–Tait equation-of-state (Eq. 4) and the bulk modulus *K*_0_ (Eq. 5). To evaluate the temperature change due to the water compression, the temperature coefficient of adiabatic compression of water (*β*_S_), defined as *β*_S_ = (∂*T*/∂*P*)_S_, is employed, where *T* is the temperature, *P* is the pressure, and *S* is the enthalpy. Then, we estimate the maximum temperature change of 3 °C at the maximum pressure change of 200 MPa at *t* = 0.2 ms in Fig. 5(f), when we use the equation of *β*_S_ = (∂*T*/∂*P*)_S_ along with the value^26^ of *β*_S_ = 0.00145 K/bar. This implies that the temperature change is negligible due to the weak compression of water.

Another estimation by ANSYS/FLUENT was done for investigating the temperature effect on the pressure and ejection velocity at the temperature of 30 °C and Run 6. In the fluid condition, the liquid density, liquid viscosity, bulk modulus at 30 °C were set to be 995.7 kg/m^3^, 0.80 × 10^−6^ m/s^2^, and 2.23 GPa, respectively. The pressure and velocity were indicated as the blue curve in Fig. 5(f). From the figure, we find that the black (20 °C case) and blue (30 °C case) curves are perfectly overlapped. This implies that the temperature difference is negligible for the pressure and ejection velocity.

### Fluid dynamic model

To investigate the high-speed liquid-jet ejection mechanism and develop a high-precision model without a discretization scheme for estimating the jet ejection velocity, we propose the use of an FDM to estimate the velocity of the ejected liquid jet generated by the PJI. The FDM is constructed using five equations, that is, the mass conservation equation (Eq. 14), Murnaghan–Tait equation-of-state (Eq. 15), a defining equation for an ejected liquid volume (Eq. 16), Bernoulli’s equation (Eq. 17), and a defining equation for the container length (Eq. 18):

14 $${\rho }_{0}{Q}_{{\rm{C}}0}={\rho }_{{\rm{C}}}(t){Q}_{{\rm{C}}}(t)+{\rho }_{0}{Q}_{{\rm{EJECT}}}(t),$$

15 $${(\frac{{\rho }_{{\rm{C}}}(t)}{{\rho }_{0}})}^{n}={(\frac{{Q}_{C0}}{{Q}_{{\rm{C}}}(t)})}^{n}=\frac{{K}_{C}(t)}{{K}_{C0}},$$

16 $${\rho }_{0}{Q}_{{\rm{EJECT}}}(t)={\int }_{0}^{\hat{t}}{\rho }_{0}{A}_{N}{U}_{N}(t^{\prime} )dt^{\prime} ,$$

17 $${P}_{C}(t)+\frac{1}{2}{\rho }_{C}(t){U}_{C}{(t)}^{2}={P}_{N}(t)+\frac{1}{2}{\rho }_{0}{U}_{N}{(t)}^{2},$$

18 $$\frac{{Q}_{C0}}{{A}_{C}}={L}_{C0}={L}_{C}(t)+{X}_{P}(t)=\frac{{Q}_{C}(t)}{{A}_{C}}+{X}_{P}(t),$$

where *ρ*_0_ is the initial liquid density, *Q*_C0_ is the initial liquid volume in the container, *ρ*_C_(*t*) is the time-dependent liquid density in the container, *Q*_C_(*t*) is the time-dependent liquid volume in the container, *Q*_EJECT_(*t*) is the ejected liquid volume, *A*_C_ is the cross-sectional area in the container, *U*_N_(*t*) is the liquid-jet ejection velocity from the nozzle, *L*_C0_ is the initial liquid length in the container (*Q*_C0_ = *A*_C_*L*_C0_), *L*_C_(*t*) is the time-dependent liquid length in the container (*Q*_C_(*t*) = *A*_C_*L*_C_(*t*)), and *X*_P_(*t*) is the piston displacement. The details of Eq. (15) are described in the Liquid Compressibility section. Here, $\hat{t}$ is the time before the one-step time Δ*t* in the time development used in the FDM calculation, that is, $\hat{t}=t-\Delta t$. Now, based on the defining equation of *K* (Eq. 5), Eq. (15) is expanded as follows:

19 $${(\frac{{\rho }_{{\rm{C}}}(t)}{{\rho }_{0}})}^{n}=\frac{{K}_{{\rm{C}}}(t)}{{K}_{0}}=1+\frac{n}{{K}_{0}}({P}_{{\rm{C}}}(t)-{P}_{0}).$$

Using Eqs. (14) and (18), we transform Eq. (19) into the following:

20 $${P}_{C}(t)={P}_{0}+\frac{{K}_{0}}{n}\{{(\frac{{\rho }_{C}(t)}{{\rho }_{0}})}^{n}-1\}={P}_{0}+\frac{{K}_{0}}{n}\{{(\frac{{Q}_{C0}-{Q}_{EJECT}(t)}{{Q}_{C0}-{A}_{C}{X}_{P}(t)})}^{n}-1\}.$$

The right-hand side of Eq. (20) is constructed using a universal constant value (*P*_0_), the physical properties of a liquid (*K*_0_, *n*), values of the container shape (*Q*_C0_, *A*_C_), input data (*X*_P_(*t*)), and the liquid ejected volume *Q*_EJECT_(*t*). When *Q*_EJECT_(*t*) is calculated using Eq. (16) iteratively, *P*_C_(*t*) on the left-hand side of Eq. (20) can be estimated using the right-hand side of Eq. (20). The values of *U*_N_(*t*) can be calculated based on *P*_C_(*t*) using Eq. (17) along with the assumptions of a *U*_C_(*t*) of ~0 m/s and *P*_N_(*t*) «*P*_C_(*t*). Therefore, *U*_N_(*t*) can be calculated in each time step using five equations (Eqs. 14–18), and we call the procedure for estimating *U*_N_(*t*) the FDM. Note that the FDM considers the variation in liquid density but neglects the effects of the liquid viscosity on the liquid-jet ejection.

The red curves in Fig. 5 show the estimated values achieved by the FDM. As the figure indicates, for a liquid-jet ejection velocity, the prediction curves (black) achieved by the discretized numerical simulation using ANSYS/FLUENT perfectly correspond to the estimated curves (red) achieved by the FDM at both the initial and the linear-motion regimes. This implies that the correspondence for the ejection velocity enables the high-speed liquid-jet velocity to be estimated by the FDM when considering the liquid density variation but neglecting the liquid viscosity. Moreover, this implies that the jet’s condition automatically changes from the compressible flow at the initial regime (e.g., *t* < 1 ms in Run 6) to the incompressible flow at the linear-motion regime (e.g., *t* > 1 ms in Run 6) and implies that the FDM can cover fluid motions at both initial and linear motion regimes. By contrast, from the figure for the liquid pressure, there is a difference between the prediction and estimation curves. The difference is large in the peak period (*t* = 0.5 ms during Run 1). The conflict relationship for the ejection velocity and pressure might be due to the hysteresis, as described below. The largest difference between the prediction and estimation curves for the liquid pressure exists at *t* = 0.5 ms, although the largest difference for the liquid-jet ejection velocity exists at *t* = 1.5 ms. That is, the high pressure at *t* = 0.5 ms may cause a high velocity at *t* = 1.5 ms, which we call the hysteresis because such a hysteresis is not included in the FDM. In other words, the hysteresis corresponds to a delay for the transformation from the pressure to the velocity. The excess pressure at *t* = 0.5 ms does not transform to the ejection velocity at that time, but rather at *t* = 1.5 ms. The excess ejection velocity is provided at *t* = 1.5 ms.

Why do we obtain good agreement between the numerical simulation and FDM, including Bernoulli’s equation? In general, Bernoulli’s equation is valid for steady, inviscid, incompressible flow. However, we can see that Bernoulli’s equation is generally used for viscous fluid flow. In this case, we can understand that the viscous effect is negligible in the fluid system. Similarly, in the present case, we also understand that both the viscous and the compressible effects are negligible in the present fluid system.

**Figure 6:**
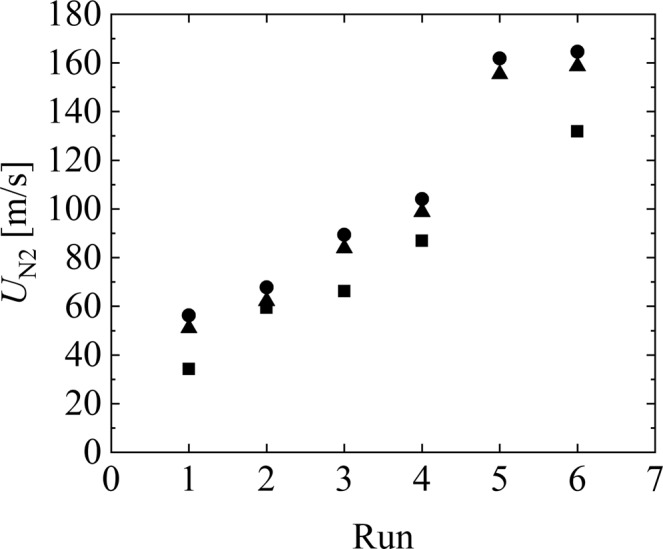
Liquid-jet ejection velocity (*U*_N2_) at *t* = 2 ms. An increment in the run number indicates the increment in the powder weight. ■, values measured by a load-cell system; ●, values predicted by ANSYS/FLUENT; and ▲, values estimated by the FDM. The 95% confidential intervals for the measured velocities were 6.5, 2.4, 4.0, 3.9, and 3.4 m/s for Runs 1–4 and 6, respectively.

Finally, we verify the predicted liquid-jet ejection velocity based on the values measured using the load-cell system. The velocity achieved by the load-cell system is calculated by Eq. (10). The velocity measured using the load-cell system is an indirect measurement value because a space of 0.5 mm exists between the needle edge and load cell. Therefore, it is difficult to compare the indirectly measured velocities with the velocities predicted by ANSYS/FLUENT only at the needle edge. In particular, the initial velocity might be strongly affected by the initial fluctuation. We then use the ejection velocity at *t* = 2 ms. Measurements were performed 3–8 times in each run. Figure 6 shows the ejection velocity (*U*_N2_) at *t* = 2 ms against each run, except for Run 5. The velocities measured by the load-cell system, predicted by ANSYS/FLUENT, and estimated using the FDM are indicated by the squares, circles, and triangles, respectively. Here, the 95% confidential intervals for the measured velocities were 6.5, 2.4, 4.0, 3.9, and 3.4 m/s for Runs 1–4 and 6, respectively. From the figure, the ejection velocities proportionally increase with increasing powder weights, although the difference between the measured and predicted velocities is 20 m/s on average. Note that the increment in the run number indicates the increment in the powder weight. This implies that (1) the numerical simulation can capture a high-speed liquid-jet ejection well, (2) a direct velocity measurement technique for a high-speed liquid jet needs to be developed in the future, and 3) the discrepancy (20 m/s) may be due to the deformation of the container and relaxation of the liquid jet after ejection.

## Conclusions

A high-speed liquid jet using a pyro jet injector (PJI) was investigated through laboratory experiments, numerical simulations, and an algebraic fluid dynamic model (FDM). The main results of this study are summarized as follows.

1. Through laboratory experiments, some of the physics and trends of the piston displacement were investigated, and the following results were found: (1) The initial and extremely rapid piston displacement of ~4 m/s might occur due to the impulsive force of the powder explosion, (2) the piston vibrates when the explosion is strong, and (3) the piston undergoes a linear uniform motion after the initial piston motion.
2. In a comparison of the discretized numerical simulations using ANSYS/FLUENT and an algebraic FDM, the liquid ejection velocity estimated by the FDM corresponds to that predicted by ANSYS/FLUENT. This indicates that the high-speed liquid-jet velocity can be estimated by the presented FDM when considering the liquid density variation but neglecting the liquid viscosity.
3. We found some characteristics of the presented PJI: (1) a very rapid piston displacement within 0.1 ms after a powder explosion, (2) the occurrence of piston vibrations only when a significant amount of powder is used, and (3) the occurrence of a pulse jet flow with a temporal width of 0.1 ms.

## References

[1] Inoue Y, Asakawa N, Hirao M (2007). High-grade food processing by water jet. Journal of Jet Flow Engineering.

[2] Adachi I (2001). Application of water-jet technique for civil engineering. Concrete Journal.

[3] Nakagawa A, Endo T, Kawaguchi T, Tominaga T (2015). Application of pulsed water jet for fine manipulation surgery. Journal of the Japan Society for Precision Engineering.

[4] Schramm J, Mitragotri S (2002). Transdermal Drug Delivery by Jet Injectors: Energetics of Jet Formation and Penetration. Pharmaceutical Research.

[5] Tagawa Y, Oudalov N, El Ghalbzoui A, Sun C (2013). Needle-free injection skin and soft matter with highly focused microjets. Lab on a Chip.

[6] Moradiafrapoli M, Marston JO (2017). High-speed video investigation of jet dynamics from narrow orifices for needle-free injection. Chemical Engineering Research and Design.

[7] Taberner A, Hogan NC, Hunter IW (2012). Needle-free jet injection using real-time controlled linear Lorentz-force actuators. Medical Engineering & Physics.

[8] Norgia M, Pesatori A (2013). Optical flow sensor through near-field grating diffraction. Sensors and Actuators A.

[9] NAKAYAMA HARUKA, PORTARO ROCCO, KIYANDA CHARLES BASENGA, NG HOI DICK (2016). CFD MODELING OF HIGH SPEED LIQUID JETS FROM AN AIR-POWERED NEEDLE-FREE INJECTION SYSTEM. Journal of Mechanics in Medicine and Biology.

[10] Li Y (1967). Equation of state of water and sea water. Journal of Geophysical Research..

[11] Gibson RE, Loeffler OH (1941). Pressure-volume-temperature relations in solutions. V. The energy-volume coefficients of carbon tetrachloride. Water and Ethylene Glycol, J. Am. Chem. Soc..

[12] Richardson JM, Arons AB, Halverson RR (1947). Hydrodynamic properties of sea water at the front of a shock wave. The Journal of Chemical Physics.

[13] Mechanical Engineering Handbook - A5 Fluid Engineering, *The Japan Society of Mechanical Engineering* (1986).

[14] Miyazaki Hiroshi, Atobe Shingo, Suzuki Takamasa, Iga Hiromitsu, Terai Kazuhiro (2019). Development of Pyro-Drive Jet Injector With Controllable Jet Pressure. Journal of Pharmaceutical Sciences.

[15] Miyazaki, H., Atobe, S., Suzuki, T., Iga, H. & Terai, K., Development of pyro-drive jet injector: Transdermal administration with the biphasic pressure delivery system and its plasmid DNA expression. 22^*nd*^*Annual Meeting of American Society of Gene & Cell Therapy*, 501, Washington (2019).

[16] Miyahara, Y. *et al*. Novel DNA vaccination utilizing a newly developed pyro-drive jet injector (PJI) induced serological and cellular immune responses leading to *in vivo* suppression of tumor growth in a rat model, 22^*nd*^*Annual Meeting of American Society of Gene & Cell Therapy*, 386, Washington, (2019).

[17] Chang, C. *et al*. Development of novel injector: pyro-drive jet injector (PJI) application to intradermal DNA vaccination, 22^*nd*^*Annual Meeting of American Society of Gene & Cell Therapy*, 497, Washington (2019).

[18] Nishikawa, T., Suzuki, S., Miki, K., Yamashita, K. & Kaneda, Y., Development of synthetic stem-loop RNA (Sl-RNA) fragment derived from sendai virus genome for inducing antitumor immunities, 22^*nd*^*Annual Meeting of American Society of Gene & Cell Therapy*, 769, Washington, (2019).

[19] Kumamaru H, Sugami H, Nakahira M, Takagaki N (2018). Particle focusing in microchannel with multi-parallel channels or porous orifice. International Journal of Mechanical Engineering and Applications.

[20] Alejandro L, Nicholls W, Stickland MT, Dempster WM (2015). CFD study of jet impingement test erosion using Ansys Fluent and OpenFOAM. Computer Physics Communications.

[21] Hemond BD, Taberner A, Hogan C, Crane B, Hunter IW (2011). Development and performance of a controllable autoloading needle-free jet injector. Journal of Medical Devices.

[22] Stachowiak JC (2007). Piezoelectric control of needle-free transdermal drug delivery. Journal of Controlled Release.

[23] Rohilla P (2019). Characterization of jets for impulsively-started needle-free jet injectors: Influence of fluid properties. Journal of Drug Delivery Science and Technology.

[24] Rohilla P, Marston JO (2019). *In-vitro* studies of jet injections. International Journal of Pharmaceutics.

[25] Shergold OA, Fleck NA, King TS (2006). The penetration of a soft solid by a liquid jet, with application to the administration of a needle-free injection. Journal of Biomechanics.

[26] Aleksandrov AA (1984). Temperature coefficient of adiabatic compression for pure water. Journal of Engineering Physics.

